# Long-term Safety and Efficacy of Belzutifan in Von Hippel–Lindau Syndrome: A VHL Coordinating Care Center Experience

**DOI:** 10.15586/jkc.v12i3.425

**Published:** 2025-09-05

**Authors:** Paulo Siqueira do Amaral, Ricardo Borges Fonseca, Breanne Reisen, Aaron Winer, Gabriel Berlingieri Polho, Robin Tumlinson, Morgan Lambrecht, Elizabeth Kaiser, Patrick David Kelly, Robert Ramirez, Daniel Barocas, Alan Tan, Kelvin Alexander Moses, Alexander Mohler, Brian Ignatius Rini, Kathryn Beckermann

**Affiliations:** 1 Department of Medicine, Vanderbilt University Medical Center, Nashville, TN, USA;; 2 Department of Radiology, Vanderbilt University Medical Center, TN, USA;; 3 Department of Neurological Surgery Nashville, TN, USA;; 4 Department of Clinical Oncology, Hospital Sirio Libânes, São Paulo, SP, Brazil;; 5 Department of Pharmacy, Vanderbilt University Medical Center, Nashville, TN, USA;; 6 Department of Urology, Vanderbilt University Medical Center, Nashville, TN, USA;; 7 Tennessee Oncology, Greco-Hainsworth Center for Cancer Research, Nashville, TN, USA

**Keywords:** Von Hippel-Lindau, Hereditary kidney cancer, Belzutifan, HIF inhibitor, safety

## Abstract

Von Hippel–Lindau (VHL) disease is a rare inherited syndrome characterized by benign and malignant neoplasms. Belzutifan, a HIF-2α inhibitor, was approved for the treatment of VHL-associated neoplasms. As a first-in-class agent, understanding tolerability and efficacy outside of a clinical trial setting and optimizing the management of adverse events (AEs) is important. We conducted a retrospective analysis of VHL patients ≥18 years old, treated with belzutifan at Vanderbilt University Medical Center, between November 2018 and December 2024. Clinical data and AEs were collected. Primary endpoint was safety; secondary endpoints included dose reduction, treatment interruption, treatment discontinuation, time to anemia onset, time to dose reduction, tumor shrinkage, objective response (per RECIST 1.1), and need for subsequent VHL-related procedures. Among 25 patients, with a median follow-up of 35.0 months, any-grade AEs occurred in 23 (92%) patients; anemia was the most frequent (64%, no grade ≥ 3). The median time to anemia onset was 3.7 months. Treatment interruption happened in 80% of the patients. The dose reduction was needed in 15 (60%) patients, with a median time of 6.8 months, and the median final dose was 80 mg. Tumor shrinkage occurred in 89% of RCC patients, 80% of CNS hemangioblastoma, and 80% of pancreatic neuroendocrine tumors (pNET). Overall, four (20%) patients experienced the progression of the disease. During follow-up, three (12%) patients required new VHL-related procedures. These findings support the long-term safety and efficacy of belzutifan in VHL disease, underscore the utility of dose reduction for AE management while demonstrating durable disease control, and a low incidence of interventional procedures.

## Introduction

Von Hippel–Lindau (VHL) disease is a rare autosomal dominant syndrome, affecting 1:36,000 people, caused by germline inactivation of the *VHL* tumor suppressor gene (1). Clinical manifestations include benign and malignant neoplasms, such as clear cell renal cell carcinoma (RCC), pancreatic neuroendocrine tumors (pNET), hemangioblastomas (HB) of the retina and central nervous system (CNS), and others (1, 2).

The loss of *VHL* function impairs the degradation of hypoxia-inducible factor (HIF), resulting in its accumulation. HIFα promotes the transcription of hypoxia-responsive genes that drive tumor development. This process contributes to the formation of angiogenic tumors, such as clear cell RCC and HB. These tumors can produce elevated levels of vascular endothelial growth factor and erythropoietin, which may further promote angiogenesis and, in some cases, lead to secondary erythrocytosis (3–5).

Historically, the management of these patients has relied on lifelong surveillance by an experienced and coordinated multidisciplinary team with regular clinical, physical, and radiologic assessments (6). Over time, a significant number of patients will require invasive interventions, which carry intrinsic risks of complications including vision loss, chronic kidney disease, and neurologic disability (7, 8).

Although VHL management has gradually improved and mortality rates have decreased, Binderup et al. estimated a mean life expectancy, lower than that of the general population, of 67 years for affected men and 60 years for women. They found that nearly 80% of the fatal events were VHL-related, mostly due to CNS HB and RCC (9).

The recent development of belzutifan, an oral HIF 2α (HIF-2α) inhibitor, represents a major advance in this field. This agent targets HIF transcription by impairing its heterodimerization with HIF-1b (ARNT), leading to durable disease control across various VHL-related neoplasms (4, 6). The LITESPARK-004 trial included 61 patients and showed an objective response rate (ORR) reaching 70% in RCC, 90% in pNET, and 50% in CNS HB, with a median duration of response not reached (10, 11). Also, it led to a significant reduction in the number of patients requiring procedural interventions (radiation or surgery) (10–12).

The prolonged time to response, from 5.5 to 11.2 months according to the type of neoplasm, highlights the importance of treatment adherence and continuous follow-up of these patients (11).

A potential lifelong therapy, the high incidence of low-grade adverse events (AEs) can impact the quality of life and the psychological burden in a young and active patient population (10). This can be illustrated by nearly one quarter of the participants discontinuing belzutifan for reasons other than progression of disease (11).

This study aims to report a single-center experience with belzutifan in patients with VHL and to provide clinical data on the safety and efficacy of this therapy at a VHL coordinating care center in an academic practice.

## Material and Methods

### Eligibility criteria

Patients aged ≥18 years with confirmed *VHL* disease who were treated with belzutifan between November 2018 and December 2024 were included.

### Data collection

A retrospective analysis was conducted at Vanderbilt University Medical Center (VUMC). Data were collected using a HIPAA-compliant registry under an Institutional Review Board–approved protocol 160979. Patients were identified through the Slicer Dicer tool within the EPIC electronic health record system by selecting individuals with a belzutifan prescription and confirmed germline *VHL* mutations.

Collected clinicopathological variables included age, sex, self-reported race, VHL-related clinical manifestations, smoking history, body mass index, and Eastern Cooperative Oncology Group (ECOG) performance status. Baseline data before belzutifan initiation included prior VHL-related interventions, complete blood count (CBC), and comprehensive metabolic panel (CMP).

After initiation of belzutifan, data collected included the start date of the treatment, initial dose, serial CBC and CMP values, documentation of any treatment interruptions, dose reductions, and discontinuation. Adverse events were captured through retrospective review of medical records. Supportive measures, including erythropoiesis-stimulating agents (ESAs), blood transfusions, and supplemental oxygen, were recorded. VHL-related interventions performed during belzutifan treatment were also captured. These included any neoplasm resection or ablation, retinal HB interventions, procedures for middle ear tumors, and phlebotomy, in one patient with erythrocytosis.

Follow-up duration was defined as the interval from belzutifan initiation until the last follow-up available or death. The date of the last data lock for this analysis was May 30, 2025.

### Assessment parameters

We evaluated the rate of AEs, categorized as any grade and grade ≥3 according to CTCAE v5.0, time to onset of anemia, rate and timing of dose reductions, rate of treatment interruptions, subsequent procedures, and proportion of treatment discontinuation.

For patients requiring dose reduction, hemoglobin (Hb) levels were assessed at three time points: at treatment initiation, at the time of dose reduction (or most recent value before dose reduction if preceded by interruption), and within 6 months post-dose reduction.

### Radiological evaluation

Radiologic assessments were performed by a neurosurgery specialist (B.R.) and an abdominal radiologist (R.B.F.). Objective response (OR) was defined according to RECIST 1.1. Baseline measurements were obtained from the most recent imaging study before belzutifan initiation, using the shortest diameter (mm). The best overall response and tumor shrinkage rate were reported separately for RCC, HB, and pNETs. Tumor shrinkage rate was defined as the proportion of patients with any decrease in tumor size relative to the total number of patients assessed per tumor type. Image intervals were based on routine clinical practice.

### Statistical analysis

Baseline characteristics were summarized using counts and percentages for categorical variables and medians with ranges for continuous variables. This was a descriptive analysis; no formal statistical hypothesis testing was performed.

## Results

### Demographic and clinical characteristics

Twenty-five patients were identified, with a median age of 42 years (range: 22 – 74), of whom 64% were females and were predominantly white (Table 1). All patients had an ECOG performance status of 0 or 1. As of May 2025, the median follow-up was 35.0 months (range: 5.6 – 79.5) after initiating belzutifan. All patients started belzutifan at 120 mg daily.

The most common VHL-associated neoplasms were CNS HB (88%), RCC (72%), retinal HB (40%) and pNET (40%). Before belzutifan initiation, 92% of patients had undergone at least one VHL-related procedure at any time point, including CNS resections (56%), nephrectomy (36%), retinal ablations (16%), pancreatic lesion resections (12%), and adrenalectomy (10%), and in one patient with erythrocytosis, serial phlebotomies. The median baseline Hb level in the entire cohort was 13.7 (range: 11.0–19.0).

**Table 1:** Baseline characteristics (n = 25).

**Age at treatment initiation – median, range**	42 (22–74)
**Gender – no. (%)**	
Female	16 (64)
Male	9 (36)
**Race – no. (%)**	
White	22 (88)
Non-white	3 (12)
**ECOG – no. (%)** 0 or 1	25 (100)
**Smoking history**	
Yes	9 (36)
No	16 (64)
**eGFR ≥ 45 mL/min – no. (%)**	25 (100)
**BMI**	
Median, range	26 (18 – 48)
< 30 – no (%)	16 (64)
≥ 30 – no (%)	9 (36)
**VHL-related neoplasms - no. (%)**	
CNS hemangioblastomas	22 (88)
RCC	18 (72)
(continues)	
Retinal hemangioblastomas	10 (40)
pNET	8 (32)
Pheochromocytoma	5 (20)
Ear endolymphatic sac tumor	2 (8)
Epididymal cystadenoma	1 (4)
**Non-neoplasm VHL manifestation**	
Erythrocytosis*	1 (4)
**Local procedure due to VHL manifestation before belzutifan – no. (%)**	23 (92)
CNS	15 (60)
Renal	9 (36)
Ophthalmologic	6 (24)
Adrenal	5 (20)
Pancreatic	4 (16)
**Baseline hemoglobin (g/dL) – median, range**	13.7 (11–19)

* For this patient, phlebotomy was considered a VHL-related intervention.

BMI, body mass index; CNS, cerebrospinal fluid; ECOG, Eastern Cooperative Oncology Group; eGFR, estimated glomerular filtration rate; pNET, pancreatic neuroendocrine tumors; RCC, renal cell carcinoma; VHL, Von Hippel–Lindau.

### Treatment safety

Adverse events were reported in 92% of patients (Table 2), with any-grade anemia being the most frequent AE (64%). No cases of Grade 3 or higher anemia were observed. The median time to anemia onset was 3.7 months (range: 1.1–33.6).

**Table 2:** Summary of adverse events and safety findings.

	Any-grade	Grade ≥ 3	Leading to dose reduction	Leading to discontinuation
Fatigue	20 (80)	1 (4)	4 (16)	0
Anemia	16 (64)	0	9 (36)	1 (4)
Nausea	8 (32)	0	2 (8)	0
Dizziness	8 (32)	0	0	0
Headache	7 (28)	0	1 (4)	0
Hypoxia	2 (8)	1 (4)	1 (4)*	1 (4)
AST or ALT elevation	2 (8)	0	1 (4)	0
Cognitive impairment	2 (8)	0	0	0
Pericardial effusion	2 (8)	1 (4)	2 (8)	0

* This patient had two subsequent dose reductions, 80 and 40 mg, without resolution of hypoxia, leading to discontinuation.

ALT, alanine aminotransferase; AST, aspartate aminotransferase.

Treatment interruptions occurred in 80% of patients (Table 2), with 28% (n = 7) experiencing interruptions lasting longer than 3 months. Of these prolonged interruptions, three were due to patient choice, two to pericardial effusion, one to fatigue, and one to loss of insurance coverage.

Dose reduction at any point was required by 60% of the patients, after a median of 6.8 months (range: 1–17), primarily due to anemia (36%), fatigue (16%), nausea (8%), and pericardial effusion (8%; Table 2). Among patients who underwent dose reduction (n = 15), median baseline Hb was 13.0 g/dL (range: 11–19). At the time of dose reduction, median Hb was 10.0 g/dL (range: 8.1–11), and after a median of 2.4 months (range: 1.2–5.5) post-reduction, the median Hb was 10.7 g/dL (range: 8.7–15.1). Following dose modification, 80% of patients experienced an increase in Hb levels. No red blood cell transfusions were required. ESAs were used in 20% of patients. One patient required supplemental oxygen due to belzutifan-induced hypoxia.

At data cutoff, 32% and 44% of the patients were on 120 and 80 mg of belzutifan daily, respectively, and 24% had discontinued treatment. The median time to discontinuation was 33 months (range: 24.5–34). Reasons for discontinuation included anemia (1 patient), Grade 3 hypoxia (1), disease progression (one pNET and one RCC), patient decision (1), and pregnancy (1).

### Efficacy in RCC, pNET, and CNS hemangioblastomas

Among the 25 patients, 23 (92%) had at least one baseline measurable target lesion according to RECIST 1.1. One patient had only nontarget CNS HB and one patient had no active neoplasm (presenting with erythrocytosis).

A total of 48 lesions were evaluated: 23 CNS HB, 17 RCC, and 8 pNET.

For RCC, nine patients were evaluated. Eight out of nine patients (89%) experienced tumor shrinkage, while one (11%) maintained tumor stability. According to RECIST 1.1, 11% had partial response (PR) and 89% had stable disease (SD). Median baseline lesion size was 24 mm (range: 19–57), and 24 mm (range: 10–53) at best response. At the data cutoff, two patients had local PD, one requiring surgery and the other with death following PD (VHL-related).

For pNET, five patients were evaluated. Median baseline size was 27 mm (range: 11–47), and 27 mm (range: 5–44) at best response. Four patients (80%) experienced tumor shrinkage, and one maintained tumor stability. Per RECIST 1.1, 20% had PR and 80% had SD. One patient had liver PD while on treatment and switched to Lu-177 dotatate.

**Figure 1:**
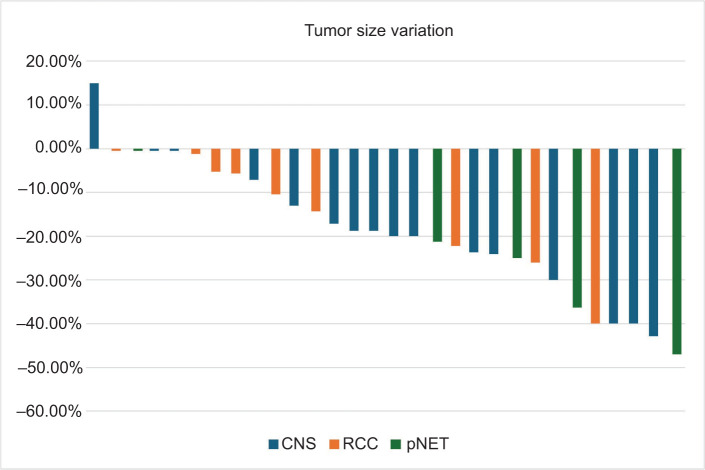
Waterfall plot showing tumor size variation at the best radiological response.

**Figure 2:**
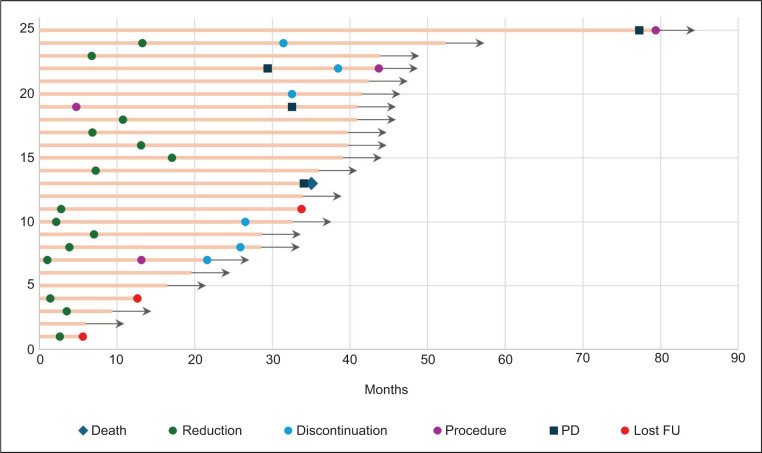
Swimmers plot showing events during follow-up.

For CNS HB, 15 patients were evaluated. The median baseline size was 14 mm (range: 10–37), and 12 mm (range: 7–37) posttreatment. Twelve patients (80%) experienced tumor shrinkage, two (13%) had stability of tumor size, and one (7%) had lesion growth. RECIST 1.1 outcomes showed 20% PR and 80% SD. At data cutoff, one patient had PD of the cystic component requiring resection (Figures 1 and 2).

Four progression events were observed (median time to progression: 36.6 months; range: 32.5–77.5), including two RCC, one CNS HB, and one pNET. All progression events occurred in patients receiving 120 mg at the time of progression; two patients continued belzutifan despite progression for perceived clinical benefit in other controlled VHL-associated lesions.

Among the 15 patients who underwent dose reduction, all patients were progression-free after a median follow-up of 27.7 months (range: 3–39) post-reduction.

### Impact on VHL-related interventions

As mentioned earlier, 92% of the patients required at least one procedure before treatment. At data cutoff, 12% of patients required new procedures related to VHL neoplasms, including two nephrectomies (one partial and one radical) and one CNS HB resection for a persistent large-volume lesion (Figure 2).

## Discussion

This academic center experience reinforces the efficacy of belzutifan in controlling VHL-associated neoplasms and highlights its generally favorable long-term safety profile in routine practice. Our findings underscore the frequent need for treatment interruptions and dose reductions outside of a clinical trial setting.

The presented cohort shares similarities with LITESPARK-004, including patient demographics and neoplasm distribution. Notably, our cohort included fewer patients with measurable (≥10 mm) RCC compared to LITESPARK-004 (36% vs 100%), as this was an inclusion criterion for the trial (12).

A similar rate of treatment discontinuation due to AEs (8%) was observed, but a higher rate of treatment interruptions (80%) and dose reductions (60%) was noted compared to LITESPARK-004 (21% and 16%, respectively) (11). These differences highlight the challenges faced in clinical practice compared to tightly controlled trial protocols. In clinical practice, in a patient population where lifelong treatment is an expectation, low-grade toxicities (e.g., headache, fatigue, nausea, anemia) may more readily prompt treatment modification.

In LITESPARK-004, nearly one quarter of the patients required ESAs for anemia, and a post-hoc analysis suggested that ESA administration did not detrimentally impact the efficacy of belzutifan (13). However, the FDA does not recommend its use since its safety has not been established (14), which shows a lack of consensus regarding the best strategy for anemia management.

Our findings suggest that dose reductions are safe and effective for anemia and improve the overall tolerability. Following dose reduction, patients maintained stable Hb levels without any indication of compromised disease control. Therefore, this approach may reduce reliance on ESAs, reduce clinic visits, financial costs, and psychological distress of self-injection, avoid ESA-related risks, and potentially mitigate other concomitant side effects.

The reasons for the differences in the objective response rates in our cohort and LITESPARK-004 are unclear. It can be attributed to several factors such as the lower number of lesions evaluated, shorter follow-up, irregular timing of radiological assessments and the larger median size of baseline lesions in our cohort. Despite these differences, our experience reinforces the efficacy of belzutifan in reducing tumor size and the need for frequent surgical interventions.

Limitations of this study include its retrospective design, single-center, and reliance on chart review to capture subjective data (e.g., adverse events) and adherence patterns.

## Conclusions

In summary, proactive toxicity management in patients treated with belzutifan is fundamental for this patient population that may require therapy for many years. Dose reduction appears to be a pragmatic approach to enhance long-term tolerability, although its potential impact on disease control warrants further study. These insights may inform personalized management strategies for VHL patients receiving belzutifan in clinical practice.

## References

[1] Nielsen SM, Rhodes L, Blanco I, Chung WK, Eng C, Maher ER, et al. Von Hippel–Lindau disease: Genetics and role of genetic counseling in a multiple neoplasia syndrome. J Clin Oncol. 2016;34(18):2172–81. 10.1200/JCO.2015.65.6140 27114602

[2] Lonser RR, Glenn GM, Walther M, Chew EY, Libutti SK, Linehan WM, et al. Von Hippel–Lindau disease. Lancet. 2003;361(9374):2059–67. 10.1016/S0140-6736(03)13643-4 12814730

[3] Haase VH. The VHL tumor suppressor: Master regulator of HIF. 2009. 10.2174/138161209789649394 PMC3622710 19671042

[4] Choueiri TK, Kaelin WG. Targeting the HIF2–VEGF axis in renal cell carcinoma. Nat Med. 2020;26(10):1519–30. 10.1038/s41591-020-1093-z 33020645

[5] Do Amaral PS, Mohan SR, Beckermann KE. Von Hippel–Lindau syndrome-related congenital polycythemia and response to belzutifan. Haematologica. 2024;109(12):4145–7. 10.3324/haematol.2024.285724 39113647 PMC11609798

[6] Daniels AB, Tirosh A, Huntoon K, Mehta GU, Spiess PE, Friedman DL, et al. Guidelines for surveillance of patients with Von Hippel–Lindau disease: Consensus statement of the International VHL Surveillance Guidelines Consortium and VHL Alliance. Cancer. 2023;129(19):2927–40. 10.1002/cncr.34896 37337409

[7] Feletti A, Anglani M, Scarpa B, Schiavi F, Boaretto F, Zovato S, et al. Von Hippel–Lindau disease: An evaluation of natural history and functional disability. Neuro Oncol. 2016;18(7):1011–20. 10.1093/neuonc/nov313 26763786 PMC4896541

[8] Capitanio U, Rosiello G, Erdem S, Rowe I, Kara O, Roussel E, et al. Clinical, surgical, pathological and follow-up features of kidney cancer patients with Von Hippel-Lindau syndrome: Novel insights from a large consortium. World J Urol. 2021;39(8):2969–75. 10.1007/s00345-020-03574-5 33416974

[9] Binderup MLM, Jensen AM, Budtz-Jørgensen E, Bisgaard ML. Survival and causes of death in patients with Von Hippel-Lindau disease. J Med Genet. 2017;54(1):11–8. 10.1136/jmedgenet-2016-104058 27539272

[10] Narayan V, Jonasch E, Iliopoulos O, Maughan BL, Oudard S, Else T, et al. Hypoxia-inducible factor-2a (HIF-2a) inhibitor belzutifan in Von Hippel–Lindau (VHL) disease-associated neoplasms: 5-year follow-up of the phase 2 LITESPARK-004 study. 2025;26(5):571–82. 10.1016/S1470-2045(25)00354-7

[11] Srinivasan R, Iliopoulos O, Beckermann KE, Narayan V, Maughan BL, Oudard S, et al. Belzutifan for Von Hippel–Lindau disease-associated renal cell carcinoma and other neoplasms (LITESPARK-004): 50 months follow-up from a single-arm, phase 2 study. Lancet Oncol. 2025;26(5):571–82. 10.1016/S1470-2045(25)00099-3 40228516 PMC12050119

[12] Jonasch E, Donskov F, Iliopoulos O, Rathmell WK, Narayan VK, Maughan BL, et al. Belzutifan for Renal Cell Carcinoma in Von Hippel–Lindau Disease. New England Journal of Medicine. 2021 Nov 25;385(22):2036–46. 10.1056/NEJMoa2103425 34818478 PMC9275515

[13] Maughan BL, Srinivasan R, Iliopoulos O, Bundsbaek A, Iversen B, Narayan V, et al. Effect of erythropoietin-stimulating agent use on belzutifan antitumor activity in patients with VHL disease-associated renal cell carcinoma: Post hoc analysis of the LITESPARK-004 study. Journal of Clinical Oncology 42(4_suppl):3–3. 10.1200/JCO.2024.42.4_suppl.3

[14] Merck Sharp & Dohme Corp. Welireg (belzutifan) [prescribing information]. Whitehouse Station (NJ): Merck Sharp & Dohme Corp.; 2021 Aug. Available from: https://www.fda.gov/drugsatfda

